# A Strictly Inducible and Orthogonal Dre-rox System for Precise and Markerless Genome Editing in *Bacillus subtilis*

**DOI:** 10.4014/jmb.2505.05006

**Published:** 2025-07-25

**Authors:** Jianan Lv, Gang Fu, Qiyao Zhu, Wenhui You, Fengming Guo, Rong Li, Dawei Zhang

**Affiliations:** 1 School of Biological Engineering, Dalian Polytechnic University, Dalian 116034, P.R. China; 2 Tianjin Institute of Industrial Biotechnology, Chinese Academy of Sciences, Tianjin 300308, P.R. China; 3 State Key Laboratory of Engineering Biology for Low-Carbon Manufacturing, Tianjin Institute of Industrial Biotechnology, Chinese Academy of Sciences, Tianjin 300308, P.R. China; 4 University of Chinese Academy of Sciences, Beijing 100049, P.R. China; 5 Tianjin University of Science and Technology, Tianjin 300457, P.R. China

**Keywords:** Dre-rox system, *Bacillus subtilis*, site-specific recombination, RNA switch regulation, orthogonal recombinase system, markerless editing

## Abstract

Site-specific recombination enables precise and modular genome engineering in microbial systems. In *Bacillus subtilis*, Cre is the most commonly used site-specific recombinase (SSR) and has been widely applied in genome engineering. Developing SSRs with comparable performance to Cre that can also function orthogonally would significantly expand the genome engineering toolkit. We established a resistance gene-based reporter in *B. subtilis* to assess the genome editing potential of the Dre-rox system. A theophylline-inducible riboswitch tightly controlled Dre expression to minimize leaky recombination, improving the specificity of rox-mediated recombination. Notably, Dre and Cre function without crosstalk at their respective recognition sites. This orthogonal combination enabled a modular workflow: Cre-mediated integration followed by Dre-mediated markerless deletion. Dual and triple-site models confirmed that Dre-rox supports synchronized multi-locus excision with a single induction. Optimized Dre-rox architecture highlighted its reliability for genome engineering in *B. subtilis*. The system features high-fidelity recombination, low toxicity, and strong host adaptability. This work extends Dre-rox utility to prokaryotic systems. The standardized Dre-rox platform provides a foundation for hierarchical pathway engineering, mutant library generation, and modular chassis development in synthetic biology.

## Introduction

Site-specific recombinases (SSRs) catalyze DNA deletions, inversions, or integrations between specific target sequences [1, 2]. SSRs are categorized as tyrosine or serine recombinases [3]. Tyrosine recombinases catalyze reversible recombination between identical sites, enabling multiple rounds of recombination [4, 5]. The well-characterized Cre recombinase from P1 phage targets 34-bp loxP sites and forms the basis of the widely used Cre-lox system [6]. Serine recombinases, *e.g.*, ΦC31 integrase, are likewise important for targeted recombination and genome editing [7, 8]. Compared to conventional strategies such as random mutagenesis or homologous recombination, SSR systems exhibit high sequence specificity and operational controllability, making them particularly suitable for conditional mutagenesis and targeted editing of small DNA fragments [9]. In recent years, with the rapid progress of synthetic biology and industrial biotechnology, increasing attention has been paid to developing high-precision genome editing tools for metabolic pathway engineering in microbial cell factories. The recombination process of SSRs usually requires only two DNA sequences carrying recombinase recognition sites and SSR proteins, does not require the involvement of cofactors or host systems, and can function normally in *vivo* and in *vitro*. Recombination systems developed based on SSRs have been used in a variety of model microorganisms [10].

The Cre-lox system remains the most extensively applied tyrosine recombinase platform in synthetic biology, each lox site comprises an 8-bp spacer flanked by two 14-bp palindromic arms. Cre recombinase mediates recombination by recognizing the 14-bp inverted repeats, cleaving the DNA, and forming a complementary interface with another cleaved lox site to complete strand exchange [11]. Continued use of the Cre-lox system may lead to the occurrence of chromosomal rearrangements when multiple loxP scars remain on the genome [2, 12, 13]. Yu *et al*. developed a Cre-lox71/66-based editing method in *Bacillus subtilis*, leaving a lox72 scar with very low Cre affinity, ensuring compatibility with subsequent lox-mediated recombination. This allows for iterative multi-gene modifications within the same strain background [14]. This iterative “insertion-deletion” editing scheme eliminates the accumulation of multiple antibiotic resistance markers, which is particularly advantageous in the engineering of industrial strains [15]. However, repeated use of the Cre-lox system reveals certain limitations: as residual lox sequences accumulate in the genome, the number of potential pseudo-lox sites increases, leading to unintended recombination events [16]; in addition, the high activity of recombinase in complex genomic contexts may cause low-frequency recombination outside of intended target sites, posing risks to chromosomal stability [17]. Sustained overexpression of Cre in mammalian cells has been reported to cause large-scale chromosomal aberrations and inhibit cell proliferation [18]. Therefore, precise spatiotemporal control of recombinase expression is usually required in practical applications to minimize host toxicity and maintain genome integrity.

**Fig. 1:**
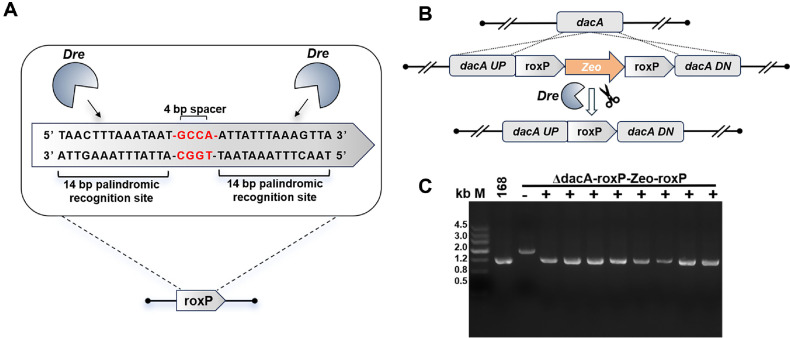
Dre-rox-mediated chromosomal excision of the Zeo resistance gene in *Bacillus subtilis*. (**A**) Schematic representation of the roxP recombination site recognized by Dre recombinase. Each roxP sequence consists of two 14 bp palindromic arms flanking a 4 bp asymmetric central spacer. Dre binds the palindromic sequences and catalyzes recombination between two roxP sites in direct orientation. (**B**) Strategy for chromosomal integration and Dre-mediated excision at the dacA locus. The roxP-Zeo-roxP cassette was integrated into the *dacA* locus. Upon IPTG induction, Dre expression was activated, leading to Zeo gene excision and leaving behind a single roxP scar. (**C**) Colony PCR analysis of *dacA*::roxP-Zeo-roxP recombination. In IPTG-induced samples (+), the expected 800 bp amplicon was observed, indicating successful excision of the Zeo cassette. In uninduced controls (-), clones retained the unexcised 1,200 bp fragment. The wild-type strain 168 was used as a negative control. M: DNA marker.

Dre, like Cre, is a P1 phage-derived tyrosine recombinase, first isolated from phage D6 [19]. It mediates recombination by specifically recognizing and cleaving the 32-bp rox site. As shown in Figure 1A the rox site consists of two 14-bp inverted repeats flanking a 4-bp spacer region. Although it shares structural similarity with the lox site, no cross-reactivity between Dre and Cre proteins on their respective target sites has been observed. This suggests the potential for orthogonal recombination using both systems in a single host [20]. Dre has been thoroughly characterized as a genome editing tool in mammalian systems and has been widely used in combination with the Cre-lox system for orthogonal lineage tracing, module tracking, and expression control in mouse models. These systems demonstrate excellent compatibility and target specificity [21-24]. In microbial cell factory construction, the Dre-rox system is often employed to excise antibiotic resistance markers from the genome, and when combined with Cre-lox, facilitates the generation of stable, plasmid-free, and markerless cyanobacterial strains [25]. However, the biosynthesis of high-value compounds often requires the development of complex, multi-tiered gene expression regulation systems, to enable spatiotemporal control of target product synthesis and support high-throughput mutant library generation, a process that frequently relies on the integration of multiple orthogonal recombination systems working in concert, to achieve multidimensional and precise rewiring of metabolic networks. An artificial combinatorial recombination platform integrating Dre-rox and other site-specific recombination systems enables large-scale and independent recombination events across various regions of the *Saccharomyces cerevisiae* genome, resulting in random deletion or inversion of target genes, which facilitates directed evolution of the yeast genome under selection pressure [4]. Although Dre recombinase holds great potential for the development of high-precision metabolic regulation systems, its utility remains constrained by the limited scope of the Dre-rox system, and few studies have reported its application in other industrial microbes. Currently, there is a gap in the application of the Dre-rox system in *B. subtilis*, and the efficiency and flexibility of genome editing in *B. subtilis* as an industrial chassis bacterium directly affect the downstream applications [14]. Introducing the Dre-rox system into *B. subtilis* may complement existing Cre-loxP tools, enabling the construction of an orthogonal dual-recombinase editing platform to facilitate more complex genome engineering tasks.

In the construction of high-fidelity recombination systems, precise regulation of recombinase expression levels is essential to ensure genetic stability and operational accuracy. On the one hand, excessive expression of recombinases may lead to cytotoxicity or off-target recombination events [26]; on the other hand, sufficient and timely expression is critical to achieving high recombination efficiency. Traditional induction systems (*e.g.*, LacI/IPTG, XylR/xylose) are widely used for prokaryotic gene expression control [27, 28], but these systems rely on protein-based regulatory elements and often suffer from leaky background expression or metabolic interference from inducers. In contrast, ligand-responsive RNA switches, which regulate downstream gene translation via aptamer-ribs-switch modules, offer unique advantages: their regulatory mechanisms are simple and rapid, requiring no additional regulatory proteins; the small size and design flexibility of RNA elements make them easy to construct and optimize in prokaryotic systems [29, 30], and they have emerged as ideal tools for improving the precision of prokaryotic expression control [31]. These systems function by embedding a ligand-binding domain within the 5' untranslated region (UTR), which undergoes RNA secondary structure rearrangement in response to the presence or absence of the inducer: in the uninduced state, steric hindrance blocks the ribosome binding site (RBS), while upon induction, conformational changes relieve the blockage, thereby enabling spatiotemporal control of target gene expression. In recent years, a variety of synthetic riboswitches have been applied to prokaryotic genetic circuit regulation, for example, the theophylline aptamer-based riboswitch has been successfully used to modulate reporter gene expression in *E. coli* and *B. subtilis* [32]. However, there have been no reports to date combining RNA switches with the Dre recombinase for genome editing. Introducing this novel post-transcriptional regulatory module into the Dre system could enable precise temporal regulation of recombinase activity, thereby ensuring editing efficiency while minimizing potential cytotoxic effects. This approach could provide a safer and more effective strategy for applying the Dre-rox system in complex genetic contexts.

Based on the above background, this study aims to evaluate and optimize a Dre-rox recombination platform suitable for the chromosomal context of *B. subtilis*. A standardized Dre expression module was constructed and tightly regulated using a theophylline-responsive RNA switch to verify its accurate excision capability across different genomic loci. Meanwhile, various rox site mutants were designed and tested, enabling systematic analysis of Dre’s sequence preference within spacer regions and its pairing-dependent recombination behavior. These analyses elucidate Dre’s recognition dependencies and orientation preferences during recombination. Finally, orthogonality with the Cre-lox system was verified, and a dual-recombinase editing strategy was developed, resulting in a highly efficient recombination platform with orthogonality, controllability, and modular compatibility. This study expands the genome engineering toolkit available for *B. subtilis*, and the proposed dual-regulated recombinase strategy offers a novel route for large-scale genome reprogramming in complex microbial chassis. It holds great promise for a wide range of synthetic biology and metabolic engineering applications.

## Materials and Methods

### Strains, Plasmids, and Culture Conditions

A complete list of strains and plasmids used in this study is provided in Table S1, and the primers used are listed in Table S2. *B. subtilis* 168 and its derived strains were used as the host chassis for chromosomal integration and phenotypic analysis in this study. All recombinant plasmids were constructed and propagated in *Escherichia coli* DH5α. Unless otherwise specified, cultures were maintained at 37°C. Solid and liquid cultures were grown on Luria-Bertani (LB) agar and LB broth, respectively, with shaking at 220 rpm to ensure proper aeration.

Antibiotics were added to the media as needed for selection. For *E. coli*, ampicillin (100 μg/ml; Macklin) was used. For *B. subtilis*, the selection was performed using 5 μg/ml chloramphenicol (Cm; Macklin), 50 μg/ml neomycin (Neo; Macklin), 100 μg/ml zeocin (Zeo; Gibco), or 100 μg/ml spectinomycin (Spe; Macklin), depending on the selection marker present. Induction of gene expression was achieved by supplementing the media with 1 mM isopropyl β-D-1-thiogalactopyranoside (IPTG), 1.5% (w/v) xylose (Macklin), or 4 mM theophylline (Macklin), according to the regulatory characteristics of the promoter used in each construct.

### Construction of Expression Vectors and Recombination Modules

The Dre recombinase expression system was engineered based on the pMA5-Pspac-lacO-Cre plasmid (designated pMD001). The coding sequence of the Dre gene (GenBank Accession No. AY753669) was commercially synthesized and incorporated into the plasmid backbone. Specifically, the primer pairs PspanK-Dre Fra.for /PspanK-Dre Fra.rev and PspanK-Dre Vec.for / PspanK-Dre Vec.rev were used to amplify the plasmid backbone and the Dre gene fragment, respectively. The resulting PCR products were purified via agarose gel electrophoresis and assembled using Gibson Assembly. The recombinant DNA was subsequently transformed into *E. coli* DH5α competent cells, yielding the plasmid pMA5-PspanK-lacO-Dre (designated pMD002).

To systematically optimize the promoter-ribosome binding site (RBS) region of pMD002, five distinct regulatory elements: Pspac-lacO, xylR-PxylA, lacO-Pspac-lacO, Pspac-RS, and Pspac-RS-lacO were PCR-amplified and directionally inserted upstream of the Dre coding sequence using Gibson Assembly. The resulting expression plasmids were introduced into *E. coli* DH5α by chemical transformation, and a panel of Dre expression vectors was successfully constructed: pMA5-Pspac-lacO-Dre (pMD003), pMA5-xylR-PxylA-Dre (pMD004), pMA5-lacO-Pspac-lacO-Dre (pMD005), pMA5-Pspac-RS-Dre (pMD006), and pMA5-Pspac-RS-lacO-Dre (pMD007).

A standardized design strategy was employed to construct a total of 25 site-specific recombination modules. Each module consisted of a core structure comprising an antibiotic resistance marker (*Zeo*, *Cm*, or *Spe*) flanked by two identically oriented recombination sites (rox or lox) (Fig. 1A). Taking the representative module *dacA*::roxP-ble-roxP as an example, three DNA fragments were individually amplified using 2×PrimeSTAR Max high-fidelity DNA polymerase (TaKaRa Bio Inc., Japan): a ~1,000 bp 5’ homology arm, a ~1,000 bp 3’ homology arm, and a central functional cassette containing the ble gene flanked by roxP sites. The recombination site sequences (rox or lox) were embedded into the primers and introduced by PCR.

The three fragments were joined via splicing by overlap extension PCR (SOE-PCR), and the assembled product was verified by 1% agarose gel electrophoresis. The correct bands were recovered using the Cycle-Pure DNA purification kit (Omega Bio-tek, USA), and the final integration constructs were stored at -20°C for downstream use.

### Chromosomal Integration and Excision Verification

Recombination validation modules were transformed into *B. subtilis* 168 competent cells using the Spizizen chemical transformation method [33]. After transformation, the cell suspensions were plated onto LB agar plates supplemented with the appropriate antibiotic and incubated at 37°C for 16 h to select positive colonies. A two-step validation procedure was employed to confirm chromosomal integration. First, colony PCR was performed to amplify the targeted integration region using specific primers (listed in Table S1). The PCR products were analyzed by 1% agarose gel electrophoresis to verify that the fragment sizes matched the expected recombination outcomes. Subsequently, the PCR products were submitted to Tsingke Biotechnology Co., Ltd., for bidirectional Sanger sequencing. The resulting sequences were aligned using SnapGene v4.2.4 to confirm the accuracy of integration at the intended genomic loci.

Verified recombinant strains were used for Dre induction experiments. Dre expression plasmids with different regulatory architectures were introduced into the target strains via the Spizizen transformation method. Following confirmation of correct recombination, the strains were reactivated in test tubes and cultured until the mid-log phase (OD_600_ = 0.4-0.6), at which point Dre expression was induced. The specific inducers used depended on the promoter type: 1 mM IPTG, 1.5% (w/v) xylose, or 4 mM theophylline (RS). After induction, cultures were streaked onto selective agar plates containing the appropriate antibiotics and incubated at 37°C for 16 h.

Single colonies were subsequently picked for PCR verification of the excision event. The targeted region was amplified, and PCR products were analyzed by 1% agarose gel electrophoresis. Successful excision was indicated by a shift in amplicon size from 1,200 bp to 800 bp. All positive PCR products were further subjected to Sanger sequencing to confirm accurate recombination at the excision sites by analyzing the sequence junctions.

### Dre Expression Toxicity and Growth Curve Analysis

Two *B. subtilis* derivative strains, 3A38 and 1A751, were selected to evaluate the cytotoxicity of Dre recombinase expression and its impact on host growth. The Dre expression plasmid pMA5-Pspac-lacO-Dre, the Cre-positive control plasmid pMA5-Pspac-lacO-Cre, and the empty vector control pMA5 were individually introduced into the host strains via the Spizizen chemical transformation method. Correct transformants were confirmed by sequencing and then cultured overnight in LB broth containing 50 μg/ml kanamycin at 37°C until the optical density at 600 nm (OD_600_) reached 6-8.

Overnight cultures were inoculated at 0.5% (v/v) into a fresh induction medium containing 2 mM IPTG in a final volume of 200 μl per well. Growth monitoring was performed in a 96-well microtiter plate (LABSELECT, 11514) using a multifunctional plate reader (BioTek Synergy Neo2) under the following conditions: 37°C incubation, orbital shaking at 731 rpm (2 mm amplitude), with automated OD_600_ readings every 30 min for 24 h. All experiments were conducted in triplicate as independent biological replicates.

### Dre-Cre Orthogonality Assessment and In Vitro Recombination

Orthogonality between the Dre and Cre recombination systems was evaluated using two independently integrated excision modules: roxP-Zeo-roxP and loxP-Cm-loxP, inserted into the *dacA* and *amyE* loci of the *B. subtilis* 168 genome, respectively. The resulting recombinant strains were transformed with either the Dre expression plasmid pMD003 or the Cre expression plasmid pMD001. Procedures for chromosomal integration and excision induction followed the methods described in Chromosomal Integration and Excision Verification.

Orthogonality was assessed using two approaches. First, colony PCR was performed with primers flanking the recombination sites, and the PCR products were analyzed by 1% agarose gel electrophoresis to detect changes in fragment length resulting from successful excision. Second, replica plating was carried out on LB agar plates containing zeocin or chloramphenicol to evaluate growth phenotypes, thereby detecting potential off-target excision events. All assays were conducted in triplicate to ensure reproducibility. For *in vitro* recombination experiments, Cre recombinase (NEB) were used.

### Agarose Gel Electrophoresis and Image Acquisition

PCR products were separated by electrophoresis on 1.0% agarose gels prepared in 0.5×TAE buffer. GoldView nucleic acid dye (Solarbio, China) was incorporated into the gel for DNA staining. After electrophoresis, DNA bands were visualized under UV transillumination, and images were captured using a gel image analysis system (Tanon MINI Space 1000).

## Results

### Chromosomal Validation of Dre-roxP Recombination and Basal Activity in *B. subtilis*

To assess the recombination activity of the Dre-rox system at the chromosomal level in *B. subtilis* 168, the Zeo resistance gene was employed as a selectable marker and inserted between two roxP sites to generate a roxP-Zeo-roxP recombination cassette. This construct was successfully integrated into the *dacA* locus of the *B. subtilis* 168 chromosome (Fig. 1B). Following transformation with plasmid pMD002 and IPTG-induced expression of Dre recombinase, no colonies were observed on zeocin-containing LB agar plates, indicating that the Zeo resistance gene was effectively excised.

Colony PCR analysis revealed that the size of the amplified fragment was reduced from 1,200 bp to 800 bp in the IPTG-induced group, consistent with the expected excision of the Zeo cassette between two direct roxP sites (Fig. 1C). Bidirectional Sanger sequencing further confirmed the presence of a single residual roxP site at the recombination junction, verifying the precision of Dre-mediated excision. These results collectively demonstrate that Dre recombinase can efficiently mediate site-specific recombination at roxP sites in the *B. subtilis* genome.

**Fig. 2:**
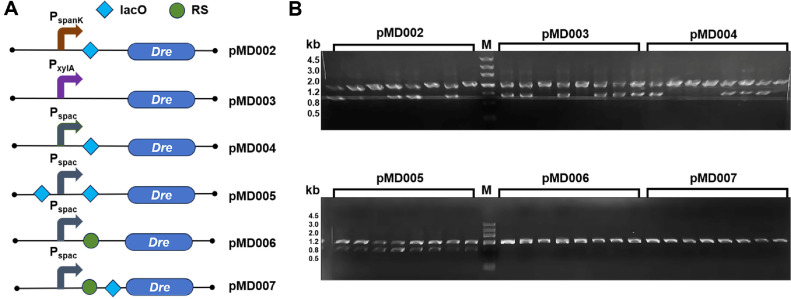
Construction and Leakiness Assessment of Dre Expression Control Systems. (**A**) Schematic representation of five Dre expression constructs with distinct regulatory modules: three protein-based regulatory systems (Pspank, PxylA, and lacO-Pspac) and two theophylline-responsive riboswitch systems (Pspac-RS and Pspac-RS-lacO), corresponding to plasmids pMD002-pMD007. (**B**) Colony PCR results under uninduced conditions. Eight colonies per construct were analyzed to detect the presence or absence of the Zeo cassette flanked by roxP sites. Leakiness was observed in traditional protein-regulated systems, as indicated by the appearance of an 800 bp deletion band. In contrast, only the intact 1,200 bp band was detected in riboswitch-regulated constructs, suggesting effective repression of Dre expression in the absence of theophylline induction. M: DNA marker.

Notably, in the uninduced control group, ~800 bp amplicons were also detected in a subset of clones, co-existing with the unexcised 1,200 bp bands. This observation suggests that Dre expression exhibits a certain degree of basal (leaky) activity even in the absence of IPTG induction (Fig. 2B).

### Dre Expression Control Optimization and Host Toxicity Assessment

To systematically address the issue of leaky Dre expression in *B. subtilis*, five regulatory architectures were constructed and comparatively evaluated. These included three conventional protein-controlled promoters (PxylA, Pspac-lacO, and lacO-Pspac-lacO) and two theophylline-responsive RNA switch systems (Pspac-RS and Pspac-RS-lacO) (Fig. 2A). Under uninduced conditions, eight individual colonies from each group were randomly selected for colony PCR analysis. The results showed that all eight colonies harboring the Pspac-lacO construct exhibited both unexcised (1,200 bp) and excised (800 bp) bands. Similarly, 6 out of 8 colonies in the PxylA group and 4 out of 8 in the lacO-Pspac-lacO group displayed dual-band patterns, indicating background recombination. In contrast, no excised bands were detected in any of the eight colonies from either RNA switch group; all exhibited a single 1,200 bp unexcised product (Fig. 2B). These findings demonstrate that the theophylline-responsive RNA switches provide more stringent repression of Dre expression under uninduced conditions compared to traditional protein-regulated systems, significantly reducing background activity and enhancing recombination fidelity.

Following the optimization of Dre expression control systems, the recombination efficiency of the RNA switch-regulated constructs was further assessed under standard induction conditions. Four individual colonies were randomly selected from each of the Pspac-RS and Pspac-RS-lacO strains and subjected to colony PCR. In all tested samples, the amplicon length was reduced from 1,200 bp to 800 bp (Fig. S1), indicating that the Zeo resistance gene was completely excised. These results confirmed that Dre recombinase maintained high excision efficiency even under tightly regulated expression and that incorporation of the RNA-based regulatory modules did not compromise the recombination functionality.

To evaluate the genetic stability of the Dre expression module in the absence of selection pressure, recombinant strains were serially inoculated in antibiotic-free LB medium for two times (48 h), followed by streaking on non-selective LB agar plates. Eight single colonies were randomly picked and analyzed by PCR using Dre-specific primers. Notably, five of the eight tested colonies no longer contained the Dre plasmid (Fig. S2), suggesting that the expression module exhibited a degree of self-curing behavior. This intrinsic instability may prove beneficial by reducing the metabolic burden on host cells and minimizing interference between genetic modules, thereby enhancing compatibility and operability during multi-round genome editing processes.

To evaluate the physiological impact of Dre expression on *B. subtilis*, Dre- and Cre-expressing strains (1A751-Dre, 1A751-Cre, 3A38-Dre, and 3A38-Cre) were constructed in the 1A751 and 3A38 genetic backgrounds. In all cases, Dre expression was controlled by the Pspac-RS promoter. Cultures were grown in an LB medium supplemented with 2 mM IPTG for 24 h, and growth curves were recorded using empty-vector strains as controls. The results showed that expression of either Dre or Cre resulted in delayed growth, with the lag phase prolonged by approximately one hour (Fig. S3A and S3B). After 24 h, the OD_600_ values of Dre-expressing strains reached 1.629 (1A751) and 1.807 (3A38), closely matching the control groups (1.649 and 1.846, respectively). In contrast, Cre-expressing strains showed slightly reduced OD_600_ values of 1.425 and 1.615 (Tables S3 and S4). indicating a more pronounced inhibitory effect. Statistical analysis confirmed that these differences were significant (*p* < 0.01). These results suggest that Dre expression may impose a slightly lower metabolic burden than Cre and potentially lead to less growth inhibition in both tested genetic backgrounds, though the observed differences are small and further studies would be needed to confirm this trend.3.3. Sequence-Dependent Recognition and Junction Specificity of Dre at Mutated rox Sites

**Fig. 3:**
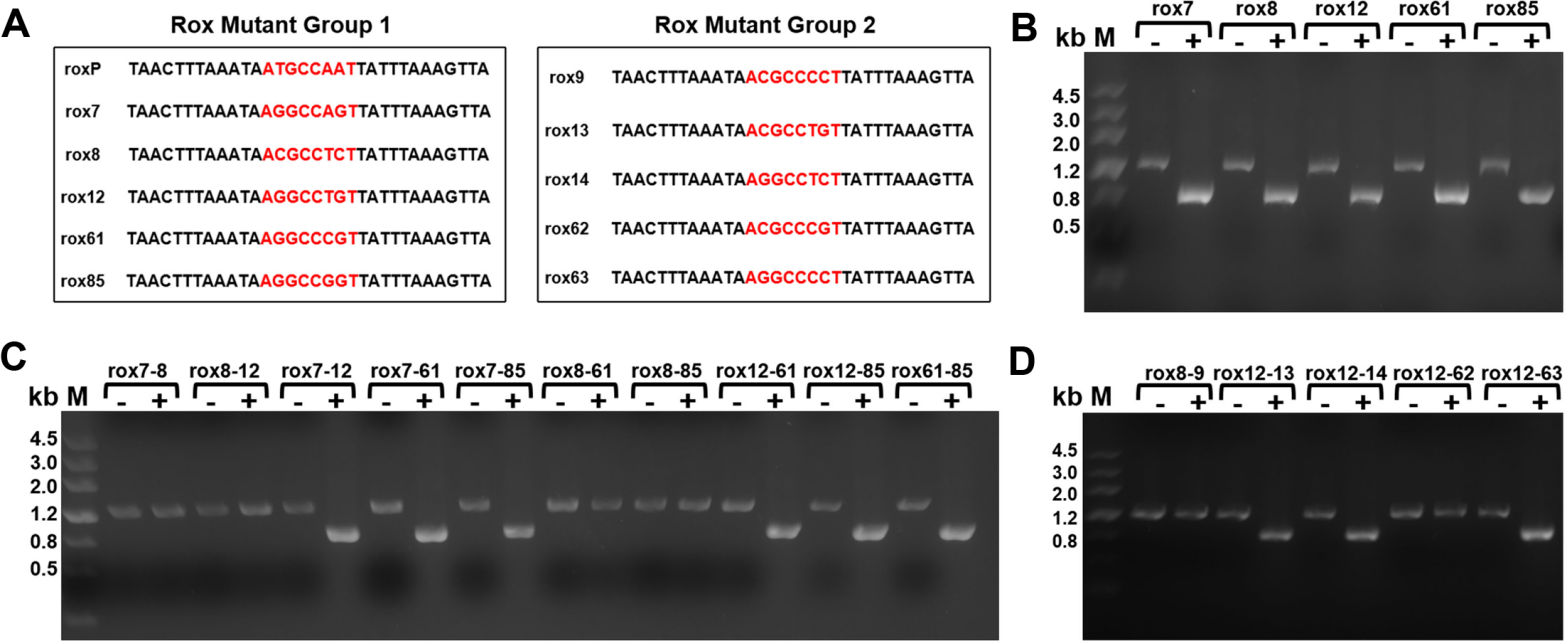
Specificity and pairing compatibility of Dre recombinase with rox site mutants in *Bacillus subtilis*. (**A**) Spacer sequences of individual rox site variants integrated into the *B. subtilis* 168 chromosome; mutated bases are shown in red. (**B**) All five single rox variants (rox7, rox8, rox12, rox61, rox85) supported Dre-mediated excision, as evidenced by a shift from 1,200 bp to 800 bp upon induction. (**B**) Pairwise recombination tests among 10 combinations of heterotypic rox sites revealed that six pairs, excluding rox8, resulted in efficient excision. (**D**) Spacer variants rox9, rox13, rox14, and rox63 (sharing 4 bp homology with active sites) supported recombination; rox62 (lacking homology) failed, indicating a 4 bp sequence dependency at the spacer region for effective Dre-mediated recombination. M: DNA marker.

To evaluate the recombination activity of Dre recombinase on mutated rox sites within the *B. subtilis* 168 genome, a series of self-recombination verification strains were constructed, each containing a Zeo resistance cassette flanked by one of five previously reported rox variants: rox7, rox8, rox12, rox61, and rox85 (Fig. 3A). These strains were transformed with the Dre expression plasmid pMD007 and induced with theophylline. PCR analysis revealed that Dre successfully mediated Zeo excision across all variants, as evidenced by a shift in amplification product size from 1,200 bp to 800 bp (Fig. 3B), demonstrating that Dre could efficiently recognize and recombine a broad spectrum of rox mutant sites. To further explore Dre's pairing specificity, ten heterologous rox site combinations were constructed (rox7-rox8, rox7-rox12, rox7-rox61, rox7-rox85, rox8-rox12, rox8-rox61, rox8-rox85, rox12-rox61, rox12-rox85, and rox61-rox85). PCR results indicated that six out of the ten combinations enabled efficient recombination, while all four combinations involving rox8 failed to recombine (Fig. 3C). Sequence alignment of the spacer regions revealed that successful rox site pairs shared at least 4 bp of homology on one side of the spacer, whereas the rox8-involved combinations lacked such homology, suggesting that Dre pairing efficiency is influenced by localized sequence compatibility within the spacer region.

To further validate the observed dependence of Dre recombinase pairing specificity on localized 4 bp sequence homology within the spacer region, five additional rox site variants were engineered. Among them, rox9 shared a 4 bp sequence with rox8 at the 5' end of the spacer region, while rox13, rox14, and rox63 each shared a 4 bp homologous sequence with rox12 at the 3' spacer end. Rox62, which had no sequence homology with rox12, was included as a negative control. Each of these rox variants was independently integrated into the *dacA* locus of the *B. subtilis* 168 genome and transformed with the Dre expression plasmid pMD007. Following induction with theophylline, colony PCR analysis showed that Dre successfully mediated Zeo excision in strains harboring the rox8-rox9 pair, as well as rox12 paired with rox13, rox14, or rox63, resulting in a reduction of PCR product size from 1,200 bp to 800 bp. In contrast, no recombination was observed in the rox12-rox62 combination (Fig. 3D). Subsequent sequence alignment confirmed that all recombinogenic rox pairs shared at least 4 bp of uninterrupted homology on one side of the spacer region, reinforcing the critical role of localized sequence conservation in Dre-mediated site pairing and recombination fidelity.

To investigate the sequence junction specificity of Dre-mediated recombination in *B. subtilis* 168, Sanger sequencing was performed on positive clones from seven successfully recombined rox site pairs: rox7-rox12, rox12-rox61, rox12-rox85, rox8-rox9, rox12-rox13, rox12-rox14, and rox12-rox63. The sequencing results consistently revealed a complementary junction pattern across all examined clones, in which one rox variant retained the 5’-terminal 4 bp of its spacer region, while the paired rox variant retained the 3’-terminal 4 bp. For instance, the recombination product of the rox7-rox12 pair preserved the 5’-4 bp from rox7 and the 3’-4 bp from rox12. Similar sequence retention patterns were observed in all other successful pairings. Importantly, no evidence of random recombination junctions or chimeric sequences was detected, confirming that Dre recombinase exhibits strict sequence selectivity in spacer-region recombination.

### Orthogonal Validation and Combinatorial Application of Cre-lox and Dre-rox Systems in *B. subtilis*

**Fig. 4:**
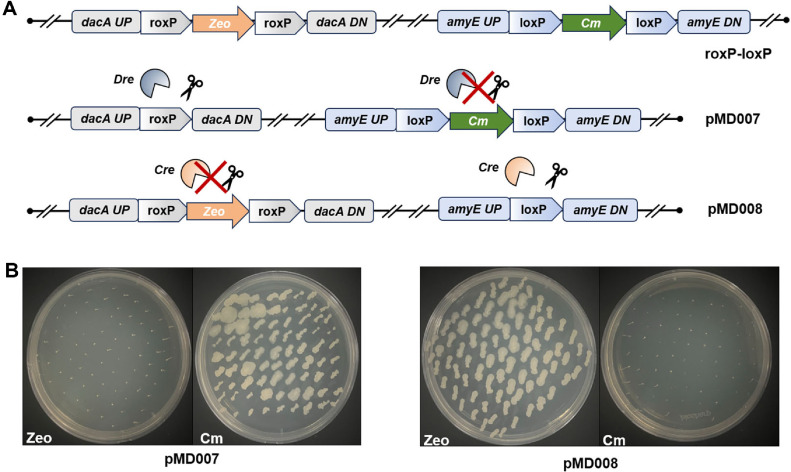
Orthogonality assessment of Dre-rox and Cre-lox systems in *Bacillus subtilis*. (**A**) Schematic of recombination modules and experimental design. Two independent reporter cassettes—roxP-Zeo-roxP and loxP-Cm-loxP-were chromosomally integrated at the *dacA* and *amyE* loci, respectively. Recombinase specificity was tested by separately introducing Dre (targeting roxP) or Cre (targeting loxP) under inducible promoters. (**B**) Phenotypic verification via replica plating. Cre-expressing strains grew only on Cm plates and lost Zeo resistance, while Dre-expressing strains grew exclusively on Zeo plates and lost Cm resistance. These results confirm the strict recombinase-site specificity and functional orthogonality of the two systems.

To address the increasing demand for orthogonal recombination systems in multiplex genome engineering, this study systematically evaluated the orthogonality between the Dre-rox and Cre-lox systems in *B. subtilis*, with a particular focus on their specificity in expression control and site recognition, as well as their potential for combinatorial application. Reporter strains were constructed by integrating either loxP or roxP sites into the genome, and then transformed with Cre or Dre expression plasmids, respectively, to generate two sets of recombination strains (Fig. 4A). Following induction under standard conditions, PCR analysis revealed that Cre-expressing strains exclusively generated loxP-specific excision bands, while Dre-expressing strains produced only roxP-specific recombination products (Fig. S4). Replica plating further confirmed these findings: under theophylline induction, Dre-expressing strains grew only on chloramphenicol-containing plates, whereas Cre-expressing strains grew solely on zeocin plates(Fig. 4B). These results demonstrate that the Dre and Cre systems exhibit strict target-site specificity and function independently without cross-reactivity, confirming their robust orthogonality within the *B. subtilis* host.

To develop a dual-system recombination platform combining Cre-lox and Dre-rox, a composite loxP-DnaK-rox12 module was constructed and chromosomally integrated into *B. subtilis* 168 using Cre recombinase-mediated circular insertion. Following verification by sequencing, the Dre expression plasmid pMD007 was introduced into the recombinant strain, and Dre expression was induced using 4 mM theophylline and 1 mM IPTG. PCR analysis of eight randomly selected colonies showed a uniform decrease in amplicon size from 4,500 bp to 4,000 bp, consistent with successful Zeo cassette excision (Fig. S5). Sanger sequencing further confirmed the precise removal of the Zeo marker and the complete retention of the DnaK open reading frame (heterologously introduced). These results collectively demonstrate that the Cre-lox and Dre-rox systems can function in concert to achieve markerless and coordinated expression of target genes within the *B. subtilis* genome.

### Evaluation of the Dre-rox System for Multi-Locus Synchronized Genome Editing in *B. subtilis*

**Fig. 5:**
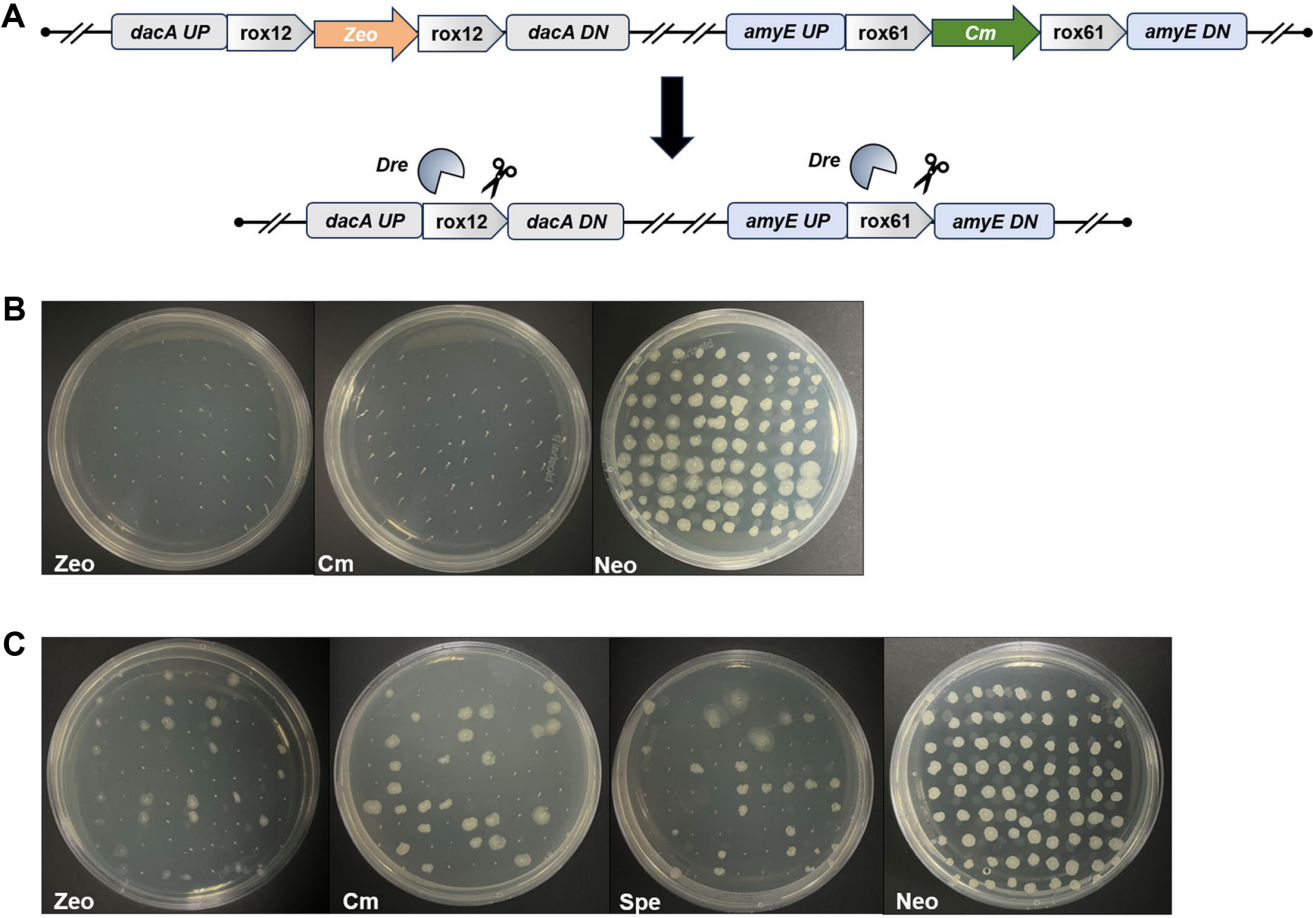
Validation of Dre-rox system adaptability for multi-site chromosomal excision in *Bacillus subtilis*. (**A**) Schematic diagram of the dual-site excision model. Recombinant modules rox12-Zeo-rox12 and rox61-Cm-rox61 were integrated into the *dacA* and *amyE* loci, respectively, to construct a dual-site synchronous excision model. (**B**) Replica plating results of the dual-site model. After theophylline induction, selected positive clones were replica plated onto Zeo and Cm plates. No colony growth was observed on either plate, while control plates showed clear colony formation, indicating successful and precise excision at both loci. (**C**) Replica plating results of the triple-site model. After additional integration of a roxP-Spe-roxP module at the *thrC* locus, positive clones were replica plated onto Zeo, Cm, and Spe plates. All colonies showed simultaneous loss of two resistance markers, but no triple-deletion clones were observed, suggesting partial limitations in triple-site coordination.

To evaluate the capacity of the Dre-rox system for simultaneous chromosomal editing at multiple loci under a single induction condition, dual-site and triple-site excision models were established in *B. subtilis* 168. In the dual-site model, two recombination cassettes, rox12-Zeo-rox12 and rox62-Cm-rox62-were independently integrated into the *dacA* and *amyE* loci, respectively (Fig. 5A). Based on this, an additional recombination cassette, roxP-spe-roxP, was inserted into the *thrC* locus to construct a triple-site model. The resulting strains were transformed with the Dre expression plasmid pMD007, and Dre-mediated recombination was induced with 4 mM theophylline. To assess the efficiency of simultaneous excision across multiple genomic loci, replica plating was performed on antibiotic selection plates corresponding to each resistance marker.

In the dual-site excision model, replica plating of 81 randomly selected single colonies revealed no growth on zeocin- or chloramphenicol-containing plates, while normal colony formation was observed on the corresponding control plates (Fig. 5B). These results indicate that the resistance markers at both the rox12 and rox62 sites were effectively excised, with a deletion efficiency approaching 100%. This demonstrates that Dre recombinase can simultaneously recognize and precisely excise both rox sites under a single induction event, exhibiting excellent efficiency and accuracy in synchronized dual-locus editing. In contrast, in the triple-site excision model, replica plating of 81 randomly selected clones showed that only two resistance markers were consistently removed, while the third remained intact, and no triple excision events were detected (Fig. 5C). This suggests that, although the Dre-rox system possesses strong multi-site recognition and excision capabilities sufficient for efficient dual-locus editing, it encounters certain limitations when applied to three-site synchronous excision. Potential contributing factors include suboptimal Dre expression levels, spatial separation of the recombination sites on the chromosome, competition among excision events, or interference with the host cell replication cycle. Further optimization and mechanistic investigation are required to enhance the applicability of the Dre-rox system in higher-order genome engineering.

## Discussion

Building upon the established application of the Dre-rox system in eukaryotic cells, this study systematically evaluated its functional adaptability and performance in the prokaryotic host *B. subtilis*. Through validation of Dre recombinase activity, optimization of expression regulation, assessment of rox site mutant recognition, verification of orthogonality with the Cre-lox system, and evaluation of multi-site excision efficiency, we developed a highly specific, low-toxicity, and modularly compatible genome recombination platform. The experimental results demonstrate that the Dre-rox system exhibits high excision efficiency and sequence fidelity in dual-locus synchronized editing, thereby providing strong support for its application in prokaryotic genome engineering.

The Dre recombinase, derived from phage D6, belongs to the tyrosine recombinase family and catalyzes site-specific excision of DNA fragments via specific recognition of rox sites [34]. In this study, by testing rox site variants, we proposed the “4 bp unilateral homology” rule, which offers experimental support for the design of rox sequences and the mechanistic interpretation of Dre-mediated recombination. The sequence fidelity and directional specificity of the recombination products were consistent with the classical Holliday junction-based recombination mechanism [35], indicating that Dre maintains stable catalytic activity within the *B. subtilis* chromosomal context. Compared to the Cre-lox system, the Dre-rox system exhibited superior biocompatibility. Previous studies have reported that Cre recombinase often induces cytotoxicity in animal cells, disrupting host physiological processes [36]. In contrast, Dre has shown no detectable toxicity in animal cells or mouse models [37].In this study, Dre caused only minor growth inhibition, likely attributable to its high target-site affinity and low non-specific binding rate, thereby reducing the metabolic burden on *B. subtilis*. This feature endows the Dre-rox system with superior adaptability compared to Cre-lox in prokaryotic genome editing.

In inducible expression systems, leaky expression under non-induced conditions is a common limitation in recombinase applications, especially during multi-round or temporally controlled genome editing processes [38]. In this study, we employed theophylline-responsive RNA switches (Pspac-RS and Pspac-RS-lacO), which regulate Dre expression by modulating RNA secondary structures, effectively suppressing its basal expression and significantly reducing unintended recombination events. Compared to traditional promoter systems, this strategy enables precise regulation at the translational initiation level, making it particularly suitable for recombinases like Dre that are sensitive to expression dosage [39, 40]. Additionally, Dre expression plasmids can be spontaneously lost during subculturing in the absence of selection pressure, simplifying the strain construction process. The high degree of orthogonality between the Dre-rox and Cre-lox systems further enhances their application flexibility. In this study, Cre-mediated in *vitro* circularization and Dre-mediated chromosomal excision were functionally decoupled, enabling a modular workflow for genome engineering. This advantage complements the precise excision capability of Dre, laying a theoretical foundation for combinatorial use with other site-specific recombinases (*e.g.*, Vika/vox, Bxb1/att) [41, 42], and expanding the toolkit for complex genome engineering.

Multi-site excision experiments demonstrated that the Dre-rox system could efficiently and simultaneously excise both the rox12 and rox62 sites in the dual-locus model, achieving an excision efficiency approaching 100%(Fig. 5B), thus confirming its reliability in moderately complex genome editing tasks. However, in the triple-locus model, no clones exhibiting simultaneous excision at all three loci were detected (Fig. 5C), suggesting that the system has limitations in handling high-complexity multi-site genome editing. This phenomenon may be attributed to insufficient Dre expression levels, limited chromosomal accessibility of certain rox sites or varying site-pair affinities. Additionally, competition among recombination events or a lack of synchronization with the host cell replication cycle may also reduce simultaneous editing efficiency. To overcome these challenges, future strategies could involve increasing Dre expression via high-copy plasmids, optimizing the chromosomal distribution of rox sites, or adjusting the timing of theophylline induction to better align with the host replication cycle. These optimizations are expected to improve the success rate of synchronous multi-locus genome editing.

Despite demonstrating the high efficiency and modularity of the Dre-rox system in *B. subtilis*, this study acknowledges several limitations. First, the detection sensitivity of replica plating may be inadequate to capture low-frequency triple-site recombination events, potentially underestimating the system’s capacity in high-order editing tasks. Second, the limited copy number of the Dre expression plasmid and the suboptimal induction conditions of the theophylline-responsive system may constrain recombination efficiency, particularly in multi-locus scenarios. Moreover, the spatial distribution of chromosomal rox sites and their interaction with host replication dynamics remain poorly characterized, which could affect recombination synchronicity. Future efforts should focus on integrating standardized synthetic biology components and automated workflows to further enhance the utility of the Dre-rox platform. For instance, coupling high-throughput screening to refine induction parameters, or integrating Dre-rox with CRISPR-Cas systems, may enable hierarchical and tunable gene regulation. Given its high sequence specificity, low cytotoxicity, and orthogonality to Cre-lox, the Dre-rox system holds substantial promise for applications in functional genomics, metabolic engineering, and the construction of complex regulatory circuits. Overall, this study establishes Dre-rox as a robust and versatile genome engineering tool for advanced strain development. Given its high efficiency, orthogonality, and the ability to achieve markerless and coordinated genome modifications, the dual recombination system established here may serve as a valuable genetic tool in industrial biotechnology. Potential applications include genome streamlining, targeted integration of metabolic pathways, or removal of antibiotic markers in food-grade microbial strains, especially in the context of enzyme production or probiotic development.

## Conclusion

This study systematically validated the recombination activity of the Dre-rox system in the *B. subtilis* genome, demonstrating its potential as an efficient and highly specific tool for prokaryotic genome editing. Experimental results showed that the Dre-rox system enabled highly efficient dual-site excision (Fig. 5B), and analysis of rox site variants led to the proposal of a “single-sided 4 bp pairing” rule, providing a theoretical basis for future site design. The use of a theophylline-responsive RNA switch effectively suppressed background expression under non-induced conditions, improving the precision of Dre expression control. The observed orthogonality between the Dre-rox and Cre-lox systems supports their application in modular editing strategies. In addition, Dre expression exerted minimal effects on host growth, suggesting suitability for multi-round strain construction. Overall, this work establishes a recombination platform compatible with *B. subtilis*, and the Dre-rox system shows practical potential for applications in genome editing, pathway optimization, and microbial strain development.

## Supplemental Materials

Supplementary data for this paper are available on-line only at http://jmb.or.kr.
