# Supplementary material for: A Strictly Inducible and Orthogonal Dre-rox System for Precise and Markerless Genome Editing in Bacillus subtilis
Source: J Microbiol Biotechnol. 2025 Jul 25;35:e2505006. doi: 10.4014/jmb.2505.05006 (PMC12331466; doi:10.4014/jmb.2505.05006)
Supplement: Supplementary file 1 [file jmb-35-e2505006-supple.pdf]

J. Microbiol. Biotechnol. 2025. 35: e2505006

https://doi.org/10.4014/jmb.2505.05006

Supplementary Tables and Figure

A Strictly Inducible and Orthogonal Dre-rox System for Precise and Markerless Genome Editing in *Bacillus subtilis*

Jianan Lv [1,2,3†], Gang Fu [2,3†], Qiyao Zhu [2,3†], Wenhui You[1,2,3], Fengming Guo[2,3], Rong Li [1*] and Dawei Zhang [2,3,4,5*]

Table S1. Strains and plasmids are used in this work.

Strain	Description	Source
E. coli DH5α	Host for construction of plasmids	Invitrogen
B.subtilis 168	wild type	This lab
168-roxP	*B.subtilis* 168 *dacA*::roxP-Zeo-roxP	This work
168-roxP-Dre	*B.subtilis* 168 *dacA*::roxP-Zeo-roxP pMD002	This work
1A751 -Dre	1A751 pMD002	This work
1A751 -Cre	1A751 pMD001	This work
3A38 -Dre	3A38 pMD002	This work
3A38 -Cre	3A38 pMD001	This work
rox7	*B.subtilis* 168 *dacA*::rox7-Zeo-rox7	This work
rox8	*B.subtilis* 168 *dacA*::rox8-Zeo-rox8	This work
rox12	*B.subtilis* 168 *dacA*::rox12-Zeo-rox12	This work
rox61	*B.subtilis* 168 *dacA*::rox61-Zeo-rox61	This work
rox85	*B.subtilis* 168 *dacA*::rox85-Zeo-rox85	This work
rox7-Dre	*B.subtilis* 168 *dacA*::rox7-Zeo-rox7 pMD007	This work
rox8-Dre	*B.subtilis* 168 *dacA*::rox8-Zeo-rox8 pMD007	This work
rox12-Dre	*B.subtilis* 168 *dacA*::rox12-Zeo-rox12 pMD007	This work
rox61-Dre	*B.subtilis* 168 *dacA*::rox61-Zeo-rox61 pMD007	This work
rox85-Dre	*B.subtilis* 168 *dacA*::rox85-Zeo-rox85 pMD007	This work
rox7-8-Dre	*B.subtilis* 168 *dacA*::rox7-8-Zeo-rox7-8 pMD007	This work
rox7-12-Dre	*B.subtilis* 168 *dacA*::rox7-12-Zeo-rox7-12 pMD007	This work
rox7-61-Dre	*B.subtilis* 168 *dacA*::rox7-61 -Zeo-rox7-61 pMD007	This work
rox7-85-Dre	*B.subtilis* 168 *dacA*::rox7-85-Zeo-rox7-85 pMD007	This work
rox8-12-Dre	*B.subtilis* 168 *dacA*::rox8-12 -Zeo-rox8-12 pMD007	This work

rox8-61-Dre	*B.subtilis* 168 *dacA*::rox8-61-Zeo-rox8-61 pMD007	This work
rox8-85-Dre	*B.subtilis* 168 *dacA*::rox8-85-Zeo-rox8-85 pMD007	This work
rox12-61-Dre	*B.subtilis* 168 *dacA*::rox12-61 -Zeo-rox12-61 pMD007	This work
rox12-85-Dre	*B.subtilis* 168 *dacA*::rox12-85-Zeo-rox12-85 pMD007	This work
rox61-85-Dre	*B.subtilis* 168 *dacA*::rox61-85-Zeo-rox61-85 pMD007	This work
rox8-9-Dre	*B.subtilis* 168 *dacA*::rox8-9-Zeo-rox8-9 pMD007e	This work
rox12-13-Dre	*B.subtilis* 168 *dacA*::rox12-13-Zeo-rox12-13 pMD007	This work
rox12-14-Dre	*B.subtilis* 168 *dacA*::rox12-14-Zeo-rox12-14 pMD007	This work
rox12-62-Dre	*B.subtilis* 168 *dacA*::rox12-62-Zeo-rox12-62 pMD007	This work
rox12-63-Dre	*B.subtilis* 168 *dacA*::rox12-63-Zeo-rox12-63 pMD007	This work
roxP-loxP	*B.subtilis* 168 *dacA*::roxP-Zeo-roxP *amyE*::loxP-Cm-loxP	This work
roxP-loxP Dre	*B.subtilis* 168 *dacA*::roxP-Zeo-roxP *amyE*::loxP-Cm-loxP pMD007	This work
roxP-loxP Cre	*B.subtilis* 168 *dacA*::roxP-Zeo-roxP *amyE*::loxP-Cm-loxP pMD008	This work
DnaK	*B.subtilis* 168::loxP-*ldh*-rox12-Zeo-rox12-DnaK-*ldh*-loxP	This work
△*dacA* △amyE	*B.subtilis* 168 *dacA*::rox12-Zeo-rox12 *amyE*::rox62-Cm-rox62 pMD008	This work
△*dacA* △amyE△thrC	*B.subtilis* 168 dacA::rox12-Zeo-rox12 *amyE*::rox62-Cm-rox62 *thrC*::loxP-Spe-loxP pMD008	This work
pMA5	A constitutive expression plasmid for *Bacillus subtilis*	This lab
pM001	pMA5-Pspac-Cre	This lab
pM002	pMA5-PspanK-lacO-Dre	This work
pM003	pMA5-Pspac-lacO-Dre	This work

pM004	pMA5-xylR-PxylA-Dre	This work
pM005	pMA5-lacO-Pspac-lacO-Dre	This work
pM006	pMA5-Pspac-RS-Dre	This work
pM007	pMA5-Pspac-RS-lacO-Dre	This work
pM008	pMA5-Pspac-RS-lacO-Cre	This work

Table S2. The primers used in this work.

Name	Sequence（5'to 3'）
dacA-rox full.for	CATTTGACAGGGTCTCTGACTCTGTC
dacA-rox full.rev	GAATCCGTAATCCTTTTCATCACCTGTGTA
dacA-rox vali.for	GATGTCATTGGTTGTGTTAACTCTAGCTGT
dacA-rox vali.rev	CGTCTACAGTCGCTTTTTATACTCGC
roxP F1.for	CTCAGTTCATTCGCATGACTGGC
roxP F1.rev	AGTAACTTTAAATAATTGGCATTATTTAAAGTTAGAATCTGCAATAGCAGCAGCATTTGCAG
roxP F2.for	AGATTCTAACTTTAAATAATGCCAATTATTTAAAGTTACTTGATATGGCTTTTTATATGTGTTACTCTACATACAGAAAG
roxP F2.rev	GACGATTCTAACTTTAAATAATTGGCATTATTTAAAGTTATCAGTCCTGCTCCTCGGCC
roxP F3.for	GCAGGACTGATAACTTTAAATAATGCCAATTATTTAAAGTTAGAATCGTCGATTGCAGAAATCGTTGCCGGTTCTGAAAC
roxP F3.rev	TTAAAACCAGCCGGTTACCGTATCA
rox7 F1.rev	AATGCTGCTGCTATTGCAGATTCTAACTTTAAATAAGGCCAGTTATTTAAAGTTA
rox7 F2.for	GCCTTATTTAAAGTTAGAATCTGCAATAGCAGCAGCATTTGCAGAATAAA
rox7 F2.rev	AAGGCCAGTTATTTAAAGTTAGAATCGTCGATTGCAGAAATCGTTGCCGGTTCTGAAA
rox7 F3.for	GACGATTCTAACTTTAAATAACTGGCCTTATTTAAAGTTATCAGTC
rox8 F1.rev	CTTTAAATAACGCCTCTTATTTAAAGTTAGAATCGTCGATTGCAGAAATCG
rox8 F2.for	CTTTAAATAAGAGGCGTTATTTAAAGTTATCAGTCCTGCTCCTCGGC
rox8 F2.rev	TTAAATAACGCCTCTTATTTAAAGTTACTTGATATGGCTTTTTATATGTGTTACTCTAC
rox8 F3.for	CTTTAAATAAGAGGCGTTATTTAAAGTTAGAATCTGCAATAGCAGCAGCATTTG
rox12 F1.rev	AGTAACTTTAAATAACAGGCCTTATTTAAAGTTAGAATCTGCAATAGCAGCAGCATTTGCAG
rox12 F2.for	AGATTCTAACTTTAAATAAGGCCTGTTATTTAAAGTTACTTGATATGGCTTTTTATATGTGTTACTCTACATACAGAAAG
rox12 F2.rev	GACGATTCTAACTTTAAATAACAGGCCTTATTTAAAGTTATCAGTCCTGCTCCTCGGCC
rox12 F3.for	GCAGGACTGATAACTTTAAATAAGGCCTGTTATTTAAAGTTAGAATCGTCGATTGCAGAAATCGTTGCCGGTTCTGAAAC

rox61 F1.rev	AGTAACTTTAAATAACGGGCCTTATTTAAAGTTAGAATCTGCAATAGCAGCAGCATTTGCAG
rox61 F2.for	AGATTCTAACTTTAAATAAGGCCCGTTATTTAAAGTTACTTGATATGGCTTTTTATATGTGTTACTCTACATACAGAAAG
rox61 F2.rev	GACGATTCTAACTTTAAATAACGGGCCTTATTTAAAGTTATCAGTCCTGCTCCTCGGCC
rox61 F3.for	GCAGGACTGATAACTTTAAATAAGGCCCGTTATTTAAAGTTAGAATCGTCGATTGCAGAAATCGTTGCCGGTTCTGAAAC
rox85 F1.rev	TAAATAACCGGCCTTATTTAAAGTTATCAGTCCTGCTCCTCG
rox85 F2.for	CTTTAAATAAGGCCGGTTATTTAAAGTTAGAATCGTCGATTGCAGAAATCGTTG
rox85 F2.rev	TTTAAATAAGGCCGGTTATTTAAAGTTACTTGATATGGCTTTTTATATGTGTTACTC
rox85 F3.for	TAAATAACCGGCCTTATTTAAAGTTAGAATCTGCAATAGCAGCAGC
rox9 F1.rev	GTAACTTTAAATAAGGGGCGTTATTTAAAGTTAGAATCTGCAATAGCAGCAGCATTTGCAG
rox9 F2.for	GCAGATTCTAACTTTAAATAACGCCCCTTATTTAAAGTTACTTGATATGGCTTTTTATATGTGTTACTCTACATACAGAAAG
rox9 F2.rev	CGACGATTCTAACTTTAAATAAGGGGCGTTATTTAAAGTTATCAGTCCTGCTCCTCGGC
rox9 F3.rev	GATAACTTTAAATAACGCCCCTTATTTAAAGTTAGAATCGTCGATTGCAGAAATCGTTGCCGGTTC
rox13 F1.rev	AGTAACTTTAAATAACAGGCGTTATTTAAAGTTAGAATCTGCAATAGCAGCAGCATTTGCAG
rox13 F2.for	AGATTCTAACTTTAAATAACGCCTGTTATTTAAAGTTACTTGATATGGCTTTTTATATGTGTTACTCTACATACAGAAAG
rox13 F2.rev	GACGATTCTAACTTTAAATAACAGGCGTTATTTAAAGTTATCAGTCCTGCTCCTCGGCC
rox13 F3.for	GCAGGACTGATAACTTTAAATAACGCCTGTTATTTAAAGTTAGAATCGTCGATTGCAGAAATCGTTGCCGGTTCTGAAAC
rox14 F1.rev	AGTAACTTTAAATAAGAGGCCTTATTTAAAGTTAGAATCTGCAATAGCAGCAGCATTTGCAG
rox14 F2.for	AGATTCTAACTTTAAATAAGGCCTCTTATTTAAAGTTACTTGATATGGCTTTTTATATGTGTTACTCTACATACAGAAAG
rox14 F2.rev	GACGATTCTAACTTTAAATAAGAGGCCTTATTTAAAGTTATCAGTCCTGCTCCTCGGCC

rox14 F3.for	GCAGGACTGATAACTTTAAATAAGGCCTCTTATTTAAAGTTAGAATCGTCGATTGCAGAAATCGTTGCCGGTTCTGAAAC
rox62 F1.rev	AGTAACTTTAAATAACGGGCGTTATTTAAAGTTAGAATCTGCAATAGCAGCAGCATTTGCAG
rox62 F2.for	AGATTCTAACTTTAAATAACGCCCGTTATTTAAAGTTACTTGATATGGCTTTTTATATGTGTTACTCTACATACAGAAAG
rox62 F2.rev	GACGATTCTAACTTTAAATAACGGGCGTTATTTAAAGTTATCAGTCCTGCTCCTCGGCC
rox62 F3.for	GCAGGACTGATAACTTTAAATAACGCCCGTTATTTAAAGTTAGAATCGTCGATTGCAGAAATCGTTGCCGGTTCTGAAAC
rox63 F1.rev	AGTAACTTTAAATAAGGGGCCTTATTTAAAGTTAGAATCTGCAATAGCAGCAGCATTTGCAG
rox63 F2.for	AGATTCTAACTTTAAATAAGGCCCCTTATTTAAAGTTACTTGATATGGCTTTTTATATGTGTTACTCTACATACAGAAAG
rox63 F2.rev	GACGATTCTAACTTTAAATAAGGGGCCTTATTTAAAGTTATCAGTCCTGCTCCTCGGCC
rox63 F3.for	GCAGGACTGATAACTTTAAATAAGGCCCCTTATTTAAAGTTAGAATCGTCGATTGCAGAAATCGTTGCCGGTTCTGAAAC
amyE-rox full.for	CAGGACCGGCGGCTGCGAGTGCTGAAA
amyE-rox full.rev	CTTCCAGGGTATGTTTCTCTTTGATGT
amyE-rox vali.for	CTGATCGATGGGATGTCACGCA
amyE-rox vali.rev	TGGCTCCAATGATTCGGATTTTGATA
loxP F1.for	GTAATCATGGTCATTATGTTTGGCGGCATCAAATCGA
loxP F1.rev	GTAAGTCTTCAAAAAATCAAATAAGGAGTGTCAAGAATG
loxP F2.for	GGATCTCCTTTTCCAGTCACGACGTTGTAAAACGAC
loxP F2.rev	GCCGCCAAACATAATGACCATGATTACGAATTCGAGCT
loxP F3.for	AAAACCCGCTCCGATTAAAGCT
loxP F3.rev	CGTCGTGACTGGAAAAGGAGATCCATTTGGCAAAACATACAC
loxP-DnaK F1.for	TTACGCTACGTATAACTTCGTATAGCATACATTATACGAAGTTATCATCAATGATGGTTTCTTTTTTGTT
loxP-DnaK F1.rev	TAACTTTAAATAACAGGCCTTATTTAAAGTTACAGGCCGCTGAATTTCCATGTTGCGTAAG
loxP-DnaK F2.for	TAACTTTAAATAAGGCCTGTTATTTAAAGTTACGATTTTCGTTCGTGAATACATGTTAT

loxP-DnaK F2.rev	AATAACAGGCCTTATTTAAAGTTATTATAATTTTTTTAATCTGTTATTTAAATAG
loxP-DnaK F3.for	TAACTTTAAATAAGGCCTGTTATTTAAAGTTAATGATCATGAACCTCACCGATAAAACCG
loxP-DnaK F3.rev	CTCTTTTGGCTAAAAGACTCCTTTTTGCTCCGTTTTGTTAT
loxP-DnaK F4.for	AAAAGGAGTCTTTTAGCCAAAAGAGCGGGTGATTGGAAGCGGCACAACA
loxP-DnaK F4.rev	CTGCACGCGTATAACTTCGTATAATGTATGCTATACGAAGTTATCGCAAGAATGCCTTTCATTTTCAAAACAGT
loxP-DnaK vali.for	GGCATCGTTAGTGAAGTCATGGC
loxP-DnaK vali.rev	GCTCTCCGATAATATGCGCGTGT
amyE-rox62 F1.for	TTCGCTTGGCTGAAAATGATTCTTCT
amyE-rox62 F1.rev	ATAACAGGCCTTATTTAAAGTTAGCCAATTTGATACGATGTCGGCTGATACAGCCAGTACC
amyE-rox62 F2.for	TAACTTTAAATAAGGCCTGTTATTTAAAGTTACGATTTTCGTTCGTGAATACATGTTATAATAAC
amyE-rox62 F2.rev	CAGTACCTAAGTAACGGTTTAACTTTAAATAACAGGCCTTATTTAAAGTTATTATAATTTTTTTAATC
amyE-rox62 F3.for	ATAACTTTAAATAAGGCCTGTTATTTAAAGTTAAACCGTTACTTAGGTACTGAACAAGAATTTAAAG
amyE-rox62 F3.rev	TGTGAGCCGCGCTGATTCATAAATATC
amyE-rox62 Full.for	GGCTTACAGAAGAGCGGTAAAAGAAG
amyE-rox62 Full.rev	GTCCAGCCATCACATTGTGAAATCT
amyE-rox62 vali.for	GAAGGATATTCATGATGCAGGATATACAGC
amyE-rox62 vali.rev	AATGAATTCTGCGTGACATCCCATC
thrC-roxP F1.for	TGCTGAGCGCCATTCCG
thrC-roxP F1.rev	GCCGTCGTTTTACTGTCAACTCAGTCAACCCTTACCG
thrC-roxP F2.for	ACTGAGTTGACAGTAAAACGACGGCCAGTGC
thrC-roxP F2.rev	CCGATTGCCCTCAGGAAACAGCTATGACCATGATTACGA
thrC-roxP F3.for	GCTGTTTCCTGAGGGCAATCGGTGATTTCTCAC
thrC-roxP F3.rev	TGAGCTTTGAAAAAATCCTTCAGCTGC
thrC-roxP Full. for	CGGCTTCGTTCATACACGGGC
thrC-roxP Full.rev	AAATGATTTGGAAGTCGTTCAAGAAGTCAA
thrC-roxP vali.for	TACGCAGTGATGTTTCCAGCATTTCCGACCG
thrC-roxP vali.rev	AGCCCGTGCTAACATGAAATGCA
PspanK-Dre Fra.for	GTGTGGCATAATGCTCGAGGGTAAATGTGAGCACTCACA
PspanK-Dre Fra.rev	GAATTAGCTTGCATGCTCATCATGAATCCATGAGACGAGAG

PspanK-Dre Vec.for	CATGGATTCATGATGAGCATGCAAGCTAATTCGGTGGAAAC
PspanK-Dre Vec.rev	CATTTACCCTCGAGCATTATGCCACACCTTGTAGATAAAGTC
PxylA-Dre Fra.for	GGCACTGAATTTGCACATGAAAAAGGAGATTTCTATTTTAGAACTC
PxylA-Dre Fra.rev	CGCTAATGATCAGTTCGGACACGTGATTTCCCCCTTAAAAATAAATTCATTCAAATACAGAT
PxylA-Dre Vec.for	GAAATCACGTGTCCGAACTGATCATTAGCGGA
PxylA-Dre Vec.rev	CTTTTTCATGTGCAAATTCAGTGCCGAATAGTCTGGACT
Pspac-Dre.for	GTGGCATAATGTGTGGAATTGTGAGCGGATAACAATTTACTAGAGTAAAGG
Pspac-Dre.rev	TCCGCTCACAATTCCACACATTATGCCACACCTTGTAGATAAAGTCAACAAC
lacO-Pspac.for	CTTGTGAGCGGATAACAACAGAACAACCTCTGCTAAAATTCCTG
lacO-Pspac.rev	GTTCTGTTGTTATCCGCTCACAAGATGTAGAACAAAACATCTTTCCGCTCTTG
Pspac-RS.for	CGTCTTGATGCCCTTGGCAGCACCCTGCTAAGGAGGAATAATTATGTCCGAACTGATCATTAGCGGATC
Pspac-RS.rev	AGGGTGCTGCCAAGGGCATCAAGACGATGCTGGTATCACCATTGCTTAATTCCTCCTTTACTCTAGTAAATTGTTATC
Pspac-RSO.for	CGTCTTGATGCCCTTGGCAGCACCTTGTGAGCGGATAACAACTGCTAAGGAGGAATAATTATGTCCGAACTGATCATTAGCGGATC
Pspac-RSO. rev	GCCAAGGGCATCAAGACGATGCTGGTATCACCATTGCTTAATTCCTCCTTTACTCTAGTAAATTGTTATCC
Dre vali.for	GATATGGTTGATGTCATGTAGCC
Dre vali.rev	GCGGTTGGGAATGTAATTCAG

Table S3. Triplicate OD_{600} Measurements for Growth Curves of 3A38.

Time (h)	3A38 OD600			+Cre OD600			+Dre OD600		
	R1	R2	R3	R1	R2	R3	R1	R2	R3
0:29:35	0.053	0.119	0.12	0.058	0.114	0.12	0.057	0.121	0.119
0:59:35	0.141	0.085	0.095	0.129	0.077	0.091	0.134	0.079	0.093
1:29:35	0.096	0.152	0.142	0.07	0.126	0.116	0.068	0.129	0.124
1:59:35	0.223	0.165	0.173	0.148	0.092	0.102	0.152	0.094	0.102
2:29:35	0.294	0.35	0.34	0.097	0.153	0.143	0.096	0.156	0.15
2:59:35	0.626	0.57	0.58	0.193	0.137	0.147	0.192	0.138	0.15
3:29:35	0.792	0.848	0.838	0.197	0.253	0.243	0.184	0.242	0.234
3:59:35	1.014	0.958	0.968	0.425	0.369	0.379	0.391	0.335	0.345
4:29:35	1.156	1.212	1.202	0.636	0.692	0.682	0.567	0.625	0.617
4:59:35	1.398	1.342	1.352	0.928	0.872	0.882	0.908	0.852	0.862
5:29:35	1.437	1.493	1.483	0.995	1.051	1.041	0.964	1.022	1.014
5:59:35	1.58	1.524	1.534	1.255	1.199	1.209	1.21	1.154	1.164
6:29:35	1.575	1.631	1.621	1.337	1.393	1.383	1.297	1.355	1.347
6:59:35	1.683	1.627	1.637	1.501	1.445	1.455	1.469	1.413	1.423
7:29:35	1.624	1.68	1.67	1.503	1.559	1.549	1.468	1.526	1.518
7:59:35	1.689	1.633	1.643	1.623	1.567	1.577	1.591	1.535	1.545
8:29:35	1.624	1.68	1.67	1.601	1.657	1.647	1.563	1.621	1.613
8:59:35	1.696	1.64	1.65	1.696	1.64	1.65	1.665	1.609	1.619
9:29:35	1.625	1.681	1.671	1.637	1.693	1.683	1.617	1.675	1.667
9:59:35	1.692	1.636	1.646	1.701	1.645	1.655	1.694	1.638	1.648
10:29:35	1.63	1.686	1.676	1.628	1.684	1.674	1.626	1.684	1.676
10:59:35	1.641	1.697	1.687	1.691	1.635	1.645	1.693	1.637	1.647
11:29:35	1.659	1.715	1.705	1.619	1.675	1.665	1.62	1.678	1.67
11:59:35	1.681	1.737	1.727	1.688	1.632	1.642	1.687	1.631	1.641
12:29:35	1.704	1.76	1.75	1.622	1.678	1.668	1.622	1.68	1.672
12:59:35	1.794	1.738	1.748	1.696	1.64	1.65	1.703	1.647	1.657
13:29:35	1.746	1.802	1.792	1.637	1.693	1.683	1.644	1.702	1.694
13:59:35	1.834	1.778	1.788	1.714	1.658	1.668	1.728	1.672	1.682
14:29:35	1.784	1.84	1.83	1.656	1.712	1.702	1.67	1.728	1.72
14:59:35	1.801	1.857	1.847	1.734	1.678	1.688	1.756	1.7	1.71
15:29:35	1.815	1.871	1.861	1.675	1.731	1.721	1.701	1.759	1.751
15:59:35	1.827	1.883	1.873	1.752	1.696	1.706	1.787	1.731	1.741
16:29:35	1.837	1.893	1.883	1.69	1.746	1.736	1.731	1.789	1.781
16:59:35	1.845	1.901	1.891	1.763	1.707	1.717	1.814	1.758	1.768
17:29:35	1.85	1.906	1.896	1.699	1.755	1.745	1.755	1.813	1.805
17:59:35	1.853	1.909	1.899	1.768	1.712	1.722	1.834	1.778	1.788
18:29:35	1.854	1.91	1.9	1.7	1.756	1.746	1.771	1.829	1.821
18:59:35	1.853	1.909	1.899	1.765	1.709	1.719	1.847	1.791	1.801
19:29:35	1.853	1.909	1.899	1.694	1.75	1.74	1.782	1.84	1.832
19:59:35	1.85	1.906	1.896	1.754	1.698	1.708	1.854	1.798	1.808
20:29:35	1.847	1.903	1.893	1.676	1.732	1.722	1.787	1.845	1.837

20:59:35	1.843	1.899	1.889	1.732	1.676	1.686	1.857	1.801	1.811
21:29:35	1.838	1.894	1.884	1.651	1.707	1.697	1.787	1.845	1.837
21:59:35	1.833	1.889	1.879	1.637	1.693	1.683	1.855	1.799	1.809
22:29:35	1.827	1.883	1.873	1.69	1.634	1.644	1.782	1.84	1.832
22:59:35	1.822	1.878	1.868	1.608	1.664	1.654	1.849	1.793	1.803
23:29:35	1.817	1.873	1.863	1.595	1.651	1.641	1.776	1.834	1.826
23:59:35	1.812	1.868	1.857	1.581	1.637	1.626	1.841	1.786	1.794

Table S4. Triplicate OD_{600} Measurements for Growth Curves of 1A751.

Time (h)	1A751 OD600			+Cre OD600			+Dre OD600		
	R1	R2	R3	R1	R2	R3	R1	R2	R3
0:29:35	0.053	0.119	0.12	0.058	0.116	0.107	0.063	0.121	0.114
0:59:35	0.138	0.082	0.092	0.127	0.075	0.089	0.133	0.081	0.095
1:29:35	0.072	0.144	0.15	0.071	0.127	0.117	0.082	0.138	0.128
1:59:35	0.209	0.151	0.159	0.172	0.114	0.122	0.19	0.132	0.14
2:29:35	0.294	0.354	0.348	0.198	0.254	0.244	0.222	0.278	0.268
2:59:35	0.588	0.522	0.522	0.412	0.356	0.366	0.438	0.382	0.392
3:29:35	0.729	0.787	0.789	0.501	0.557	0.547	0.519	0.575	0.565
3:59:35	0.962	0.896	0.898	0.766	0.71	0.72	0.768	0.712	0.722
4:29:35	1.101	1.159	1.151	0.777	0.833	0.823	0.788	0.844	0.834
4:59:35	1.292	1.236	1.246	0.998	0.942	0.952	0.991	0.935	0.945
5:29:35	1.342	1.4	1.392	1.063	1.119	1.109	1.071	1.127	1.117
5:59:35	1.487	1.431	1.441	1.242	1.186	1.196	1.222	1.166	1.176
6:29:35	1.49	1.548	1.54	1.301	1.357	1.347	1.264	1.32	1.31
6:59:35	1.622	1.566	1.576	1.479	1.423	1.433	1.437	1.381	1.391
7:29:35	1.596	1.654	1.646	1.49	1.546	1.536	1.463	1.519	1.509
7:59:35	1.701	1.645	1.655	1.614	1.558	1.568	1.623	1.567	1.577
8:29:35	1.652	1.71	1.702	1.593	1.649	1.639	1.605	1.661	1.651
8:59:35	1.762	1.696	1.706	1.68	1.624	1.634	1.695	1.639	1.649
9:29:35	1.701	1.759	1.751	1.612	1.668	1.658	1.661	1.717	1.707
9:59:35	1.79	1.734	1.744	1.691	1.635	1.645	1.739	1.683	1.693
10:29:35	1.741	1.799	1.791	1.635	1.691	1.681	1.668	1.724	1.714
10:59:35	1.822	1.766	1.776	1.678	1.622	1.632	1.681	1.737	1.727
11:29:35	1.75	1.808	1.8	1.585	1.641	1.631	1.667	1.723	1.713
11:59:35	1.832	1.766	1.776	1.652	1.596	1.606	1.735	1.679	1.689
12:29:35	1.756	1.814	1.806	1.567	1.623	1.613	1.683	1.739	1.729
12:59:35	1.837	1.771	1.781	1.632	1.576	1.586	1.679	1.735	1.725
13:29:35	1.754	1.812	1.804	1.545	1.601	1.591	1.676	1.732	1.722
13:59:35	1.825	1.759	1.769	1.605	1.549	1.559	1.691	1.747	1.737
14:29:35	1.745	1.803	1.795	1.529	1.585	1.575	1.685	1.741	1.731
14:59:35	1.82	1.754	1.764	1.591	1.535	1.545	1.693	1.749	1.739
15:29:35	1.751	1.809	1.801	1.512	1.568	1.558	1.692	1.748	1.738
15:59:35	1.802	1.746	1.756	1.584	1.528	1.538	1.694	1.75	1.74
16:29:35	1.721	1.779	1.771	1.504	1.56	1.55	1.695	1.751	1.741
16:59:35	1.79	1.734	1.744	1.577	1.521	1.531	1.693	1.749	1.739
17:29:35	1.716	1.774	1.766	1.495	1.551	1.541	1.699	1.755	1.745
17:59:35	1.775	1.719	1.729	1.553	1.497	1.507	1.699	1.755	1.745
18:29:35	1.702	1.76	1.752	1.478	1.534	1.524	1.687	1.743	1.733
18:59:35	1.764	1.708	1.718	1.542	1.486	1.496	1.688	1.744	1.734
19:29:35	1.684	1.742	1.734	1.467	1.523	1.513	1.677	1.733	1.723
19:59:35	1.75	1.694	1.704	1.527	1.471	1.481	1.666	1.722	1.712
20:29:35	1.673	1.731	1.723	1.453	1.509	1.499	1.669	1.725	1.715

20:59:35	1.729	1.673	1.683	1.511	1.455	1.465	1.654	1.71	1.7
21:29:35	1.654	1.712	1.704	1.437	1.493	1.483	1.645	1.701	1.691
21:59:35	1.716	1.66	1.67	1.496	1.44	1.45	1.637	1.693	1.683
22:29:35	1.639	1.697	1.689	1.422	1.478	1.468	1.624	1.68	1.67
22:59:35	1.699	1.643	1.653	1.479	1.423	1.433	1.62	1.676	1.666
23:29:35	1.622	1.68	1.672	1.409	1.465	1.455	1.605	1.661	1.651
23:59:35	1.683	1.627	1.636	1.459	1.403	1.414	1.595	1.651	1.64

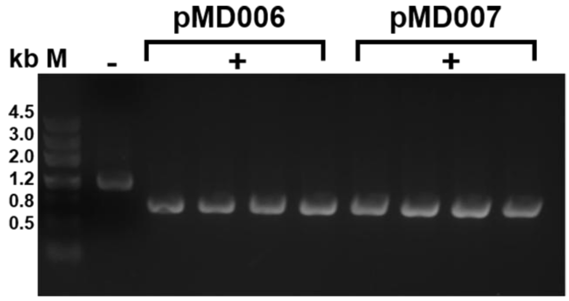

Figure S1. Validation of Dre-mediated recombination under induced conditions using tightly controlled expression systems. Four single colonies were randomly selected from *Bacillus subtilis* strains carrying the Dre expression plasmids pMD006 (Pspac-RS) or pMD007 (Pspac-RS-lacO), and subjected to colony PCR under standard induction conditions (4 mM theophylline for pMD006, and 4 mM theophylline plus 1 mM IPTG for pMD007). The results showed a uniform 800 bp deletion band in all induced samples, whereas only the uncut 1200 bp band was detected in the uninduced groups. No nonspecific bands were observed. These results indicate that Dre maintains robust recombination activity under tightly regulated expression conditions. M: DNA marker. “- and “+” indicate uninduced and induced state.

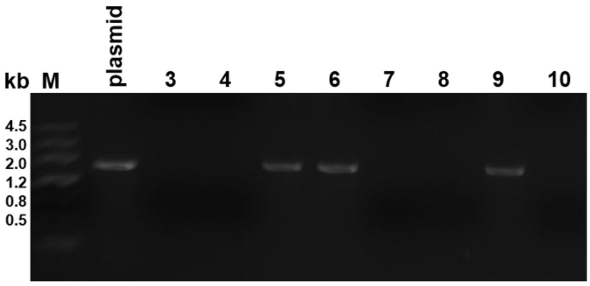

Figure S2. Stability analysis of Dre expression plasmid under non-selective conditions. To assess the plasmid stability of Dre expression constructs, the recombinant strains were inoculated for two times (48 h) in LB broth without antibiotics and streaked onto non-selective LB agar plates. Eight single colonies were randomly selected and subjected to PCR using plasmid-specific primers. As shown in the figure, Stability of the Dre expression plasmid in B. subtilis under non-selective conditions. Lane 2: positive control showing the 1800 bp Dre plasmid-specific band. Lanes 3-10: colony PCR of eight randomly picked clones. Bands were detected in Lanes 5, 6 and 9, indicating plasmid retention; no bands were observed in the remaining lanes, indicating plasmid loss. M: DNA marker.

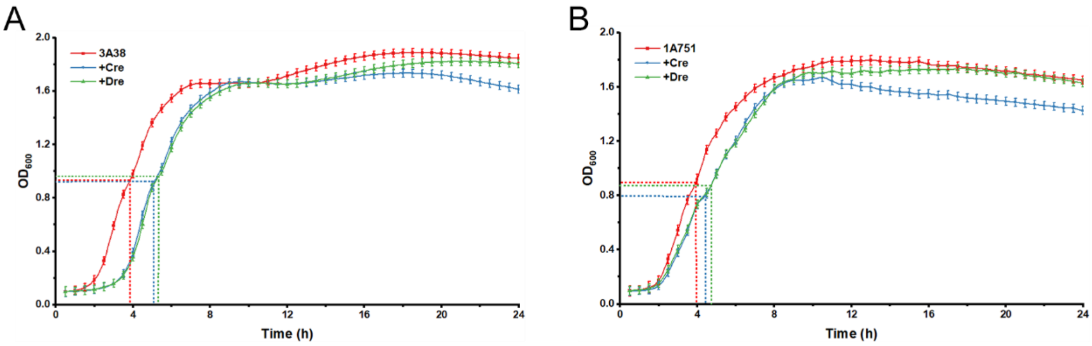

Figure S3. Growth curve analysis of *Bacillus subtilis* expressing Dre or Cre recombinase. Growth kinetics of Dre- and Cre-expressing *Bacillus subtilis* strains were assessed in two genetic backgrounds (3A38 and 1A751). Strains harboring Dre or Cre expression plasmids were cultured in LB medium supplemented with 2 mM IPTG, and OD600 values were measured every 30 minutes in 96-well plates. Control strains (Ctrl) contained empty vectors. The left panel shows the growth curves of 3A38 derivatives, and the right panel displays the results for 1A751 derivatives. Data represent means ± standard deviation (SD) from three independent biological replicates. Statistical significance was determined by t-test. Significant differences were observed between 3A38-Dre and 3A38-Cre strains, as well as between 1A751-Dre and 1A751-Cre strains ($P < 0.01$).Full replicate data are provided in Tables S3 and S4.

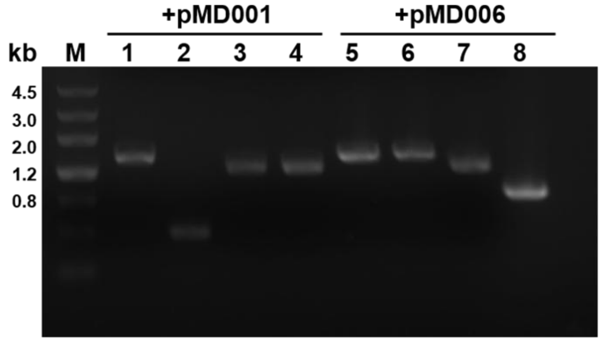

Figure S4 PCR-based verification of site-specific recombination by Cre and Dre recombinases in *Bacillus subtilis*. A dual-site recombination reporter strain harboring amyE::loxP-Cm-loxP and dacA::roxP-Zeo-roxP was constructed and transformed with either the Cre expression plasmid pMD008 or the Dre expression plasmid pMD007. After induction under standard conditions, site-specific recombination events were analyzed by colony PCR.

Lanes 1-4 represent the Cre expression group: lanes 1 and 3 show uninduced controls for the loxP (1500 bp) and roxP (1200 bp) loci, respectively; lane 2 shows a 500 bp excision product at the loxP site upon Cre induction; lane 4 remains at 1200 bp, indicating no excision at the roxP site. Lanes 5-8 represent the Dre expression group: lanes 5 and 7 are uninduced controls at the loxP and roxP loci, respectively; lane 6 remains uncut at 1500 bp; lane 8 shows a 400 bp excision product at the roxP site following Dre induction. M: DNA marker.

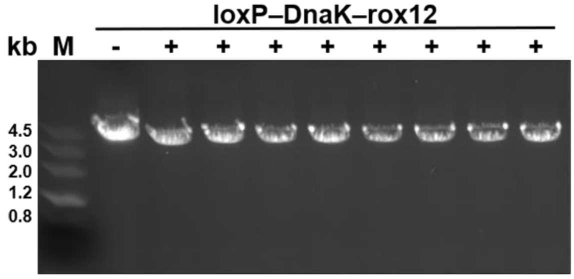

Figure S5 PCR verification of Dre-mediated site-specific excision at the *ldh* locus in *Bacillus subtilis*. A loxP-DnaK-rox12 cassette was integrated into the *ldh* locus via Cre-mediated recombination to establish a dual-recombinase expression framework. The resulting strain was subsequently transformed with the Dre expression plasmid pMD007 and induced with theophylline to activate Dre recombinase. Colony PCR was performed on eight randomly selected clones under induced (“+”) and uninduced (“-”) conditions. All induced samples showed a shift in amplicon size from 1200 bp to 800 bp, corresponding to successful Dre-mediated excision between the two rox12 sites. No nonspecific bands were detected, indicating high excision fidelity and high specificity of recombinase activity. M: DNA marker.
